# A configurational approach to leadership behavior through archetypal analysis

**DOI:** 10.3389/fpsyg.2022.1022299

**Published:** 2023-01-13

**Authors:** Janka I. Stoker, Harry Garretsen, Dimitrios Soudis, Tim Vriend

**Affiliations:** ^1^Department of HRM & OB, Faculty of Economics and Business, University of Groningen, Groningen, Netherlands; ^2^Department of GEM, Faculty of Economics and Business, University of Groningen, Groningen, Netherlands; ^3^International Business School, Hanze University of Applied Sciences, Groningen, Netherlands

**Keywords:** person-oriented approach, archetypal analysis, configurations, leadership styles, archetypes

## Abstract

The behavioral approach to leadership, which has introduced leadership styles, has been of great importance to the leadership field. Despite its importance, scholars have recently argued and demonstrated that these styles have various conceptual, methodological, and empirical limitations that could hamper further development of the leadership field. Consequently, they have called for alternative approaches to study leadership. We argue that taking a configurational or person-oriented approach to leadership behavior, which focuses on ideal-type configurations of leadership behaviors to identify leadership archetypes, offers such an alternative. We demonstrate the potential of such an approach *via* the use of archetypal analysis, for a dataset of 46 behaviors across 6 leadership styles, including more than 150,000 respondents. Our results offer a clear indication for the existence of archetypes of leadership. We also suggest how the resulting archetypes can get a meaningful interpretation, and discuss implications for future research.

## 1. Introduction

The introduction of the behavioral approach to leadership has greatly advanced our understanding of leader effectiveness (House and Aditya, 1997; Judge et al., 2004; Yukl, 2013). The behavioral approach advocates that leadership is best understood by deconstructing leaders into, various, separate leadership styles. Since the introduction of this approach, scholars have been measuring leadership behaviors *via* a range of leadership styles. Examples are the classic styles “initiating structure” and “consideration” (Stogdill, 1950) or transactional and transformational leadership (Burns, 1978), but also more recently developed styles like authentic, ethical, and servant leadership (Dansereau et al., 2013; Dinh et al., 2014).

Research shows that leadership styles relate to several leader and follower outcomes (e.g., Judge et al., 2004; Judge and Piccolo, 2004; Van Dierendonck et al., 2014; Banks et al., 2016). Meta-analytic studies also indicate that leadership styles are crucial predictors of leader effectiveness (Judge et al., 2004; Judge and Piccolo, 2004; Ceri-Booms et al., 2017; Banks et al., 2018). Furthermore, different leadership styles add unique variance in explaining leader effectiveness (DeRue et al., 2011). Accordingly, both research and practice have studied extensively how the adoption of distinct leadership styles can maximize the effectiveness of (future) leaders (Avolio et al., 2009; Day et al., 2014).

But despite their importance and prevalence, scholars have identified various conceptual, methodological, and empirical limitations of such well-established behavioral leadership styles (Yukl, 1999; Van Knippenberg and Sitkin, 2013; Fischer and Sitkin, 2022). These limitations include a substantial empirical overlap both within and between these styles (Judge and Piccolo, 2004; Avolio and Gardner, 2005; Van Dierendonck et al., 2014; Banks et al., 2016), a lack of integration (Yukl, 1999; Van Knippenberg and Sitkin, 2013; Antonakis and House, 2014), and a focus on individual rather than configurations of styles (Yukl, 2012). The substantial empirical overlap across different leadership styles also makes theory building unnecessarily complex (Banks et al., 2016).

The meta-analysis of DeRue et al. (2011) shows a significant amount of empirical overlap between different leadership behaviors, leading the authors to conclude: “given the empirical similarities between leader behaviors found in this study, we encourage scholars to develop new or revised existing measures of leader behaviors such that we can better capture the conceptual distinctions among leader behaviors” (DeRue et al., 2011, p. 38). Banks et al. (2018) used meta-analytic correlations to confirm that construct redundancy remains problematic for the field of leadership. Next to construct redundancy, and based on a comprehensive assessment of the 10 most prominent leadership styles, Fischer and Sitkin (2022) conclude that the main problem of these leadership styles is that they all mix “the description of the content of leadership behaviors with the evaluation of their underlying intentions, quality of execution, or behavioral effects” (2022, p. 1). Their proposed way forward is to study leadership styles in a configurational manner.

The discussion about conflated leadership styles in leadership research relates to a fundamental debate in organizational and psychological science at large (Fiss, 2007; MacDougall et al., 2014; Bogat et al., 2016; Foti and McCusker, 2017), which is the distinction between a variable-and a person-oriented approach (Block, 1971; Bergman and Magnusson, 1997; Foti and Hauenstein, 2007; Scheuer et al., 2022). A variable-oriented approach assumes that the respective variables are different from each other. For leadership research, such an approach is problematic because the variables concerned, namely leadership styles, show a strong overlap. Also, and almost by definition, every person and thus leader is imperfectly defined by just a set of distinct leadership styles. In this sense, the call for a person-oriented approach where the person is the focal point, seems especially relevant for the field of leadership (Foti et al., 2012; Foti and McCusker, 2017; Scheuer et al., 2022). More generally, this call fits in what has been labeled as a “paradigm shift” in organizational research, because the person-oriented approach truly offers a new and different approach of investigating organizational questions (Woo et al., 2018).

Our paper aims to show how to use such a configurational or person-oriented approach and to which actual configurations it gives rise. We do so by using a large dataset of more than 150,000 respondents and 46 behaviors of leaders. We build configurations by applying archetypal analysis. Archetypal analysis is a classification method in which “archetypes” can be derived from combined behavioral configurations. This method is related to the pattern approach to leadership that takes the individual leader as holistic entity (Fiss, 2007; Foti et al., 2012; Scheuer et al., 2022) as the focal point of the analysis. So, our specific research question to answer is whether we can arrive, through archetypal analysis, at meaningful configurations of leadership behaviors.

Our configurational approach by means of archetypal analysis leadership behaviors offers three contributions. First, by applying archetypal analysis to a large data set that encompasses more than 150,000 managers across multiple countries, we respond to the call for a configurational or person-oriented approach in the leadership field. In doing so, we offer an alternative to study leadership behavior that addresses the conceptual and empirical limitations of current behavioral approaches (Fischer and Sitkin, 2022). Second, we show that, at least in our sample, the resulting archetypes are distinct from (the sum of) the separate underlying leadership styles, and thereby that an archetype indeed can offer a more holistic and at the same time more comprehensive perspective on leadership than separate leadership styles (Fiss, 2007; Foti et al., 2012; Fischer and Sitkin, 2022). Such a holistic approach seems particularly useful in times, like the current ones, where the degree of volatility and uncertainty confronting organizations and their managers is substantial. Instead of relying and using an ever-increasing number of fixed leadership styles, a different and more flexible perspective is needed to characterize leader behavior, which is a call for more agile or adaptive leadership approaches. Third, by applying the method of archetypal analysis, we empirically illustrate how our configurational approach can indeed lead to conceptually meaningful leadership archetypes. Such archetypes can be helpful to guide leaders and their organizations in contexts that are highly volatile, uncertain, complex and ambiguous.

In the remainder of this paper, we will first explain the characteristics of a configurational approach to leadership. Next, we will briefly discuss what archetypes and archetypal analysis are. We are then in a position to empirically demonstrate the potential use of archetypal analysis for the field of leadership, by using a dataset of 46 behaviors across 6 leadership styles. We find evidence for three archetypes, and these resulting archetypes can be defined along two main dimensions in our view. By means of a regression analysis we show that these archetypes are related to, but crucially also are distinct from, the underlying six leadership styles. The paper ends with a conclusion and discussion section, and presents avenues for future research.

## 2. Theoretical framework

### 2.1. Configurations of leadership

Conceptually, the call for the development of possible leadership configurations is not new among leadership scholars (see Fleishman et al., 1991; Yukl, 1999; Morgeson et al., 2010). Already in the 1960s, Blake and Mouton (1964) introduced the so-called Managerial Grid, which is based on the idea that there are five “types” of leaders, based on the combination of two seminal leadership styles, namely concern for task and concern for people. Also, the idea of so-called leadership “archetypes” has been suggested before in the leadership literature by leadership scholars, see for instance Kets de Vries et al. (2010) and Yukl (2013).

Nevertheless, one of the key features of virtually all leadership studies is that they investigate one or only a very few leadership styles. This approach raises two main concerns. First, by reducing individuals to one or more separate leadership styles, these studies fail to acknowledge that leaders can display various behaviors simultaneously (Yukl, 2012). Second, we also know that considering leadership styles in isolation may yield invalid estimates (Antonakis et al., 2010; Antonakis and House, 2014), with seemingly strong and reliable effects potentially disappearing when controlling for multiple leadership styles (e.g., Judge and Piccolo, 2004; Banks et al., 2016).

Given these two concerns, scholars have called for a critical investigation into the use of current leadership styles (Yukl, 1999; Dansereau et al., 2013; Van Knippenberg and Sitkin, 2013; Antonakis and House, 2014; Banks et al., 2016; Fischer and Sitkin, 2022), and for using alternative approaches to study leadership behavior. As argued by Fischer and Sitkin (2022), studying configurations of leadership is one way to address these concerns. Configurations reduce theoretical complexity, by integrating overlapping and separate leadership styles (Fleishman et al., 1991), thereby leaving room for a more parsimonious analysis that is focused on the core elements of the resulting configuration (Fiss, 2007; Delbridge and Fiss, 2013; Snow and Ketchen, 2014). Moreover, it allows to effectively and efficiently consider more intricate leadership behaviors that would be difficult to establish by considering only (the interaction between) individual leadership styles.

To date, there have been a few attempts to empirically arrive at configurations of leadership behavior. O’Shea et al. (2009) studied leadership patterns for the combination of transformational and transactional leadership. In their paper, predefined patterns are developed based on the combination of these leadership styles, leading to typical “high-high” or “high-low” combinations (see also Scheuer et al., 2022). In a similar vein, Arnold et al. (2017) applied a person-oriented approach to the same leadership styles. However, these studies use a pattern analysis which is still based on the combination of a few styles, and not on all the underlying individual behaviors. This last observation is also true for the pattern analysis of (self and ideal) leadership perceptions in Foti et al. (2012), where confirmatory factor analysis and latent profile analysis are employed in order to find patterns of individual traits in self and ideal leader profiles.

We thus need other approaches to reach meaningful leadership configurations. When it comes to the actual configurations employed, and without being restricted to a pre-determined limited set of leadership styles, we propose that, compared to other clustering techniques, archetypal analysis is such a promising approach. Primarily this is the case because archetypal analysis offers a configurational approach that is not based on some averaging technique of groups or clusters of observations, but instead on actual individual leadership behaviors instead of leadership styles in the data set. Before we turn to our actual application, we will first elaborate on the characteristics of archetypal analysis.

### 2.2. Empirically classifying archetypes through archetypal analysis

Archetypal analysis characterizes observations in a data set as convex combinations of extremal points, which allows all other observations to be described as a mixture of these extremal points. An everyday analogy is the phrase “There’s a little bit of _____ in everyone.” Given a dataset, archetypal analysis identifies those unique characteristics, the mixture of which makes up all the observed types, and estimates the proportion of all characteristics in each observation.

Thus, archetypal analysis shares characteristics with commonly used clustering techniques as well as dimensional reduction techniques like principal components. But it differs from these more standard clustering techniques such as cluster analysis or factor analysis, in a sense that archetypal analysis identifies different archetypes in the data based on extreme behavioral configurations of individuals (here, leaders). It determines for each individual case how close this individual is to each of these extreme archetypes, and is therefore a novel example of a configurational approach. Whereas these more standard data reduction techniques focus on the similarity between groups of observations, archetypal analysis emphasizes the boundaries of a data set. In layman’s terms, archetypal analysis allows to identify different archetypes based on extreme individual behavioral configurations, and assigns a proximity score to these archetypes for each individual observation. In doing so, archetypal analysis is a prime example of the configurational or person-oriented approach (Foti and McCusker, 2017).

In addition to the advantages of ideal-type configurations as mentioned above, the data-driven approach of archetypal analysis enables the identification of archetypes without a theoretical prior, which is advantageous given that the field of leadership is thus characterized by a plethora of different, overlapping theoretical models and meta-categories (Van Knippenberg and Sitkin, 2013; Banks et al., 2016, 2018; Fischer and Sitkin, 2022). Archetypal analysis already has been used in various academic fields to identify patterns of functional vision loss (Elze et al., 2015), extreme performers (Porzio et al., 2008), usage and assessment of online courses (Kazanidis et al., 2016), published scientists (Seiler and Wohlrabe, 2013), and extreme climate and weather patterns over time (Steinschneider and Lall, 2015). In an international business setting, archetypal analysis has been used to analyze patterns of multiple cultural dimensions (Venaik and Midgley, 2015; Richter et al., 2016; De Wit, 2021), Building on these recent applications and the developments they have sparked in their respective fields, we therefore introduce archetypal analysis to the field of leadership research by applying archetypal analysis to leadership behavior.

## 3. Methodology

### 3.1. Design and sample

We make use of a large, existing dataset collected by the international consulting firm Korn Ferry (for more information on the dataset, see Euwema et al., 2007; Van Emmerik et al., 2010). The data collection was part of the assessment that took place before the start of the management training programs provided by this firm within each of the participating organizations, which guaranteed a response rate of approximately 100% (see Euwema et al., 2007). The data nowadays are collected fully online, whereas 20 years ago a combination of paper, fax and “teleform” (via score forms) was common.

Data on leadership behaviors were collected from both managers and their subordinates. We only use the subordinate ratings of leadership behavior, because it is well-known that the use of self-ratings of leadership is problematic (Harris and Schaubroeck, 1988; for an overview see Fleenor et al., 2010). We included only those countries with 500 managers or more. This led to a total sample of more than 150,000 managers from 38 countries (see Table 1), and 23 types of sectors. By far the most important sectors are manufacturing, professional services, pharmaceuticals, financials, and not-for profit/government. For each country, the leadership behaviors were translated into the language of the country, including variations, such as French–French but also Canadian French, US and US English, Brazilian Portuguese, et cetera.

**Table 1:** Sample composition.

Country	*N*	Percent
United States	36.080	13,5
United Kingdom	19.826	7,4
China	11.796	4,4
Brazil	9.536	3,6
Australia	7.467	2,8
India	6.612	2,5
Netherlands	5.862	2,2
Japan	4.867	1,8
Mexico	4.793	1,8
Germany	4.433	1,7
Malaysia	4.418	1,6
France	3.715	1,4
Poland	2.978	1,1
South Korea	2.973	1,1
Columbia	2.864	1,1
Spain	2.861	1,1
Italy	2.427	0,9
New Zealand	1.985	0,7
Belgium	1.883	0,7
Canada	1.630	0,6
Chile	1.608	0,6
Turkey	1.499	0,6
Ireland	1.421	0,5
South Africa	1.386	0,5
Singapore	1.258	0,5
Venezuela	1.231	0,5
Argentina	987	0,4
Peru	886	0,3
Somalia	876	0,3
Portugal	825	0,3
Russia	816	0,3
Slovakia	796	0,3
Sweden	772	0,3
Thailand	769	0,3
Czechia	731	0,3
Indonesia	632	0,2
Egypt	591	0,2
Philippines	536	0,2
	156.626	

### 3.2. Research instrument

#### 3.2.1. Leadership behaviors

Each leader is rated by approximately five subordinates on a total of 46 questions across six behavioral leadership styles: authoritative, affiliative, coaching, participative, directive, and pacesetting leadership (see for more information about these scales, see Euwema et al., 2007; Wendt et al., 2009; Van Emmerik et al., 2010). Although these leadership styles were developed by the consulting firm itself (based on Litwin and Stringer, 1968; Tagiuri and Litwin, 1968), they strongly resemble existing approaches, such as the directive, achievement-oriented, participative, and supportive leader behaviors as specified by path-goal theory (*cf*. House, 1971). All items used Likert-type scales, with answers ranging from 1 to 6, with alternative answers on the extreme poles. For each manager, the scores of on average five subordinates were aggregated. We examined the justification for aggregating subordinates’ responses by calculating the ICC (1) value for each leadership style (James, 1982).

*Authoritative leadership* can be defined as a leadership style where the leader exercises control, and where the underlying intent is to promote employees’ welfare. As a result, employees understand that the rules are there for their own benefit. Consequently, they respect the leader’s decisions and comply with the rules (Pellegrini and Scandura, 2008). The scale consists of nine items and an example item is: “My manager often gives orders in the form of a suggestion, but makes it clear what he/she wants” (alpha = 0.84, ICC = 0.23).

*Affiliative leadership* is closely linked to the concept of “consideration” and can be defined as degree to which a leader shows concern and respect for followers, looks out for their welfare, and expresses appreciation and support (Bass, 2008). The scale consists of eight items and an example item is: “My manager often demonstrates concern for subordinates” (alpha = 0.87, ICC = 0.28).

*Coaching leadership* can be defined as behavior oriented towards the development of employees (Stoker et al., 2001; Bass, 2008). The scale consists of six items and an example item is: “My manager puts a great deal of effort into developing subordinates” (alpha = 0.85, ICC = 0.25).

*Participative leadership* can be defined as delegation of responsibilities, and shared influence in decision-making (e.g., Somech, 2005, 2006). The scale consists of seven items and an example item is: “Encourages subordinates to participate in most decision making” (alpha = 0.68, ICC = 0.23).

*Directive leadership* is aimed at giving clear and detailed directions to followers, structuring tasks and expecting compliance with instructions (see, e.g., House, 1971; Somech, 2006; Kamphuis et al., 2011; Lorinkova et al., 2013). The scale consists of nine items and an example item is: “Expects employees to follow his/her instructions precisely” (alpha = 0.81, ICC = 0.38).

*Pacesetting leadership* is behavior in which the leader shows that he/she expects excellence and self-direction (see, e.g., Goleman, 2000). The scale consists of seven items and an example item is: “As long as my manager sees results, he/she does not get involved in subordinates’ work” (alpha = 0.68, ICC = 0.27).

*Controls*. The data also provide information on the individual leader level, as well as on the country level. For the individual leader, we control for age, gender, educational level, tenure, and nationality. At the country level, we control for national culture. We follow the 11 country-clusters as defined by Ronen and Shenkar (2013). We include these variables because previous research based on the same underlying data set has shown that these variables are related to leadership behavior (see Stoker et al., 2019; Garretsen et al., 2022).

## 4. Results

Our archetypal analysis consists of two steps. We first investigate whether it is possible to extract configurations of leadership behavior *via* archetypal analyses. Second, based on the results, and by way of illustration, we try to give meaning to the resulting archetypes.

### 4.1. Identifying archetypes

**Figure 1:**
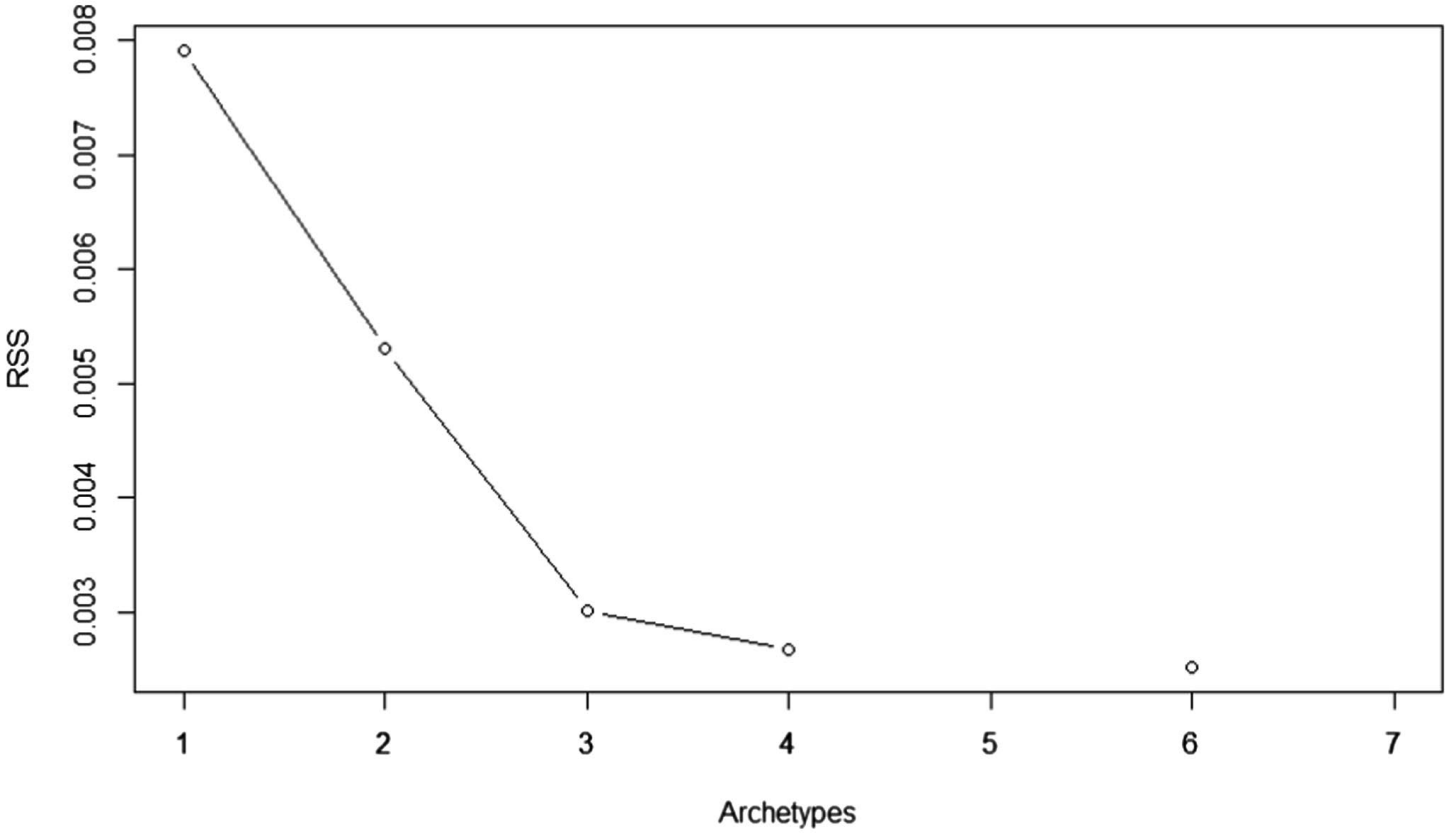
Screen plot of residual sum of squares.

Even though archetypal analysis focuses on individual behaviors and not on averages, the actual application of the archetypal technique is rather similar to other clustering approaches. The phases of the application are (1) fitting the model to the data, (2) deciding on the number of archetypes to retain, and (3) look at items’ configurations within the extracted archetypes. To determine the number of archetypes to retain, we fitted the model using 1–10 archetypes, and looked at the resulting Residual Sum of Squared Errors (RSS) in a screen plot. The optimal number of archetypes to retain was three, because the addition of extra archetypes did not significantly reduce the RSS (see Figure 1).

**Figure 2:**
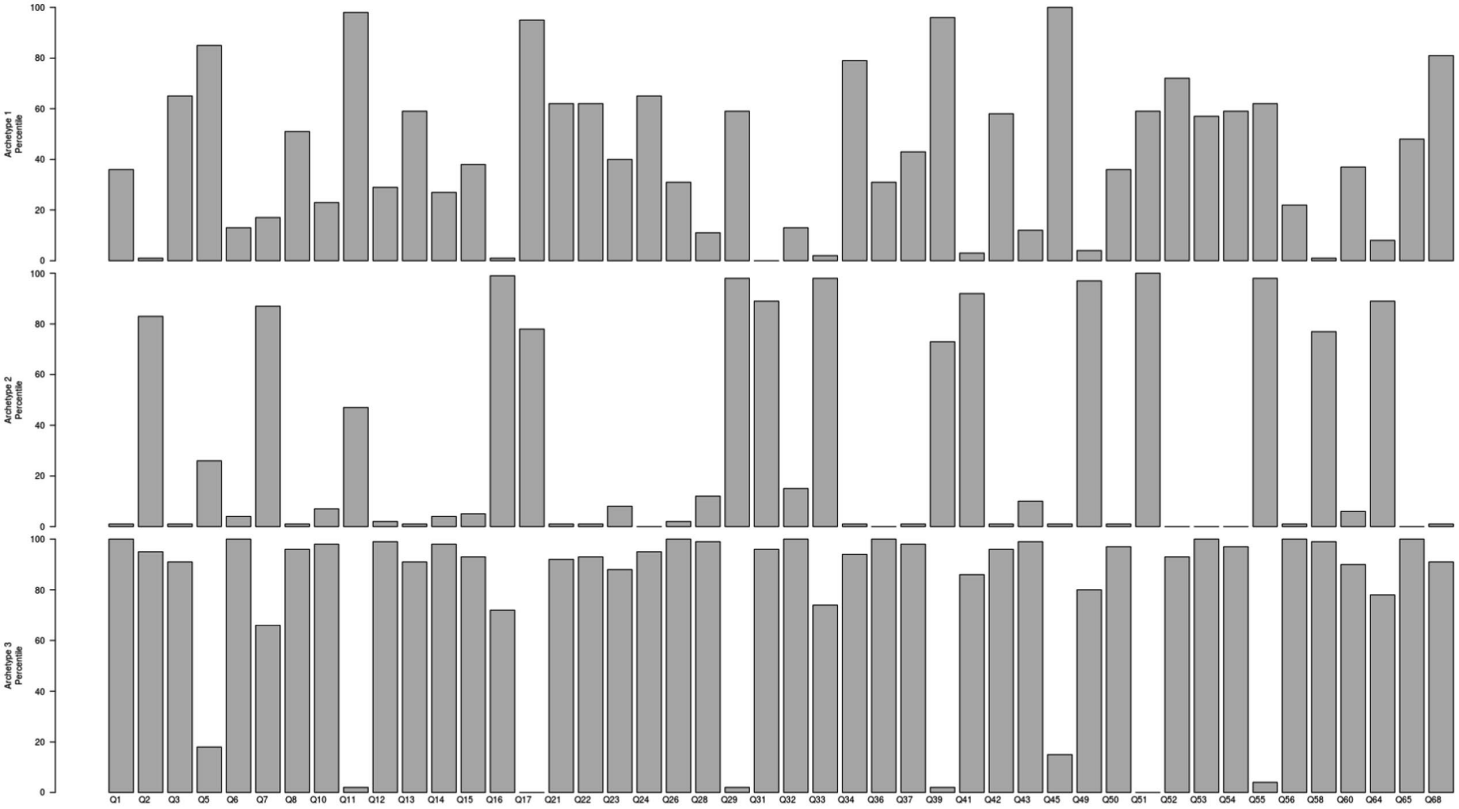
Behavioral score distributions for the three archetypes.

Based on the three archetypes in Figure 1, Figure 2 shows the barplots of the distributions of the leaders’ ratings across the three archetypes for each of the 46 items. Each archetype is characterized by a set of questions that reflect the different behaviors measured by the survey. The height of the bars in Figure 2 signifies the association between the archetype and the behavior measured by the question. For each archetype, we only include items with relatively high scores, that is above the 80th percentile. It is evident that item scores above this threshold of the 80th percentile display strong associations with that particular archetype, as compared to the other two archetypes (Eugster and Leisch, 2009).

**Figure 3:**
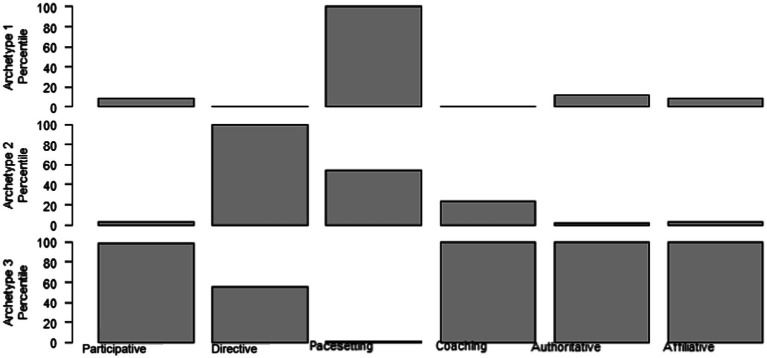
The relationship between the three archetypes and the six leadership styles.

In order to be able to interpret the possible content of the archetypes, we show for each item to which archetype the item belongs. These results are presented in Table 2. The first observation based on Table 2 as well as Figure 2, is that the archetypes are not a straightforward combination of any of the six leadership styles. The same conclusion can be drawn from Figure 3, which also illustrates that each of the archetypes is a combination of different behaviors belonging to the six leadership styles, but crucially, that they not simply match the leadership styles.

**Table 2:** 46 Behavioral questions used to classify leaders, along with their archetype association.

Archetype 1
To instruct subordinates, my manager relies primarily on providing an example through his/her own behavior (Q5)
As long as my manager sees results, he/she does not get involved in subordinates´ work (Q11)
My manager does not ordinarily check on subordinates´ progress until their assigned tasks are due (Q17)
My manager expects subordinates to figure out for themselves how to do their jobs (Q39)
My manager gives capable subordinates the freedom to make decisions and mistakes without close supervision (Q45)
Archetype 2
My manager requires subordinates to submit detailed reports of their activities (Q2)
My manager makes sure that he/she does the important tasks himself/herself (Q7)
My manager makes most decisions for subordinates (Q16)
When a subordinate’s work begins to fall short, my manager takes over the task himself/herself (Q29)
My manager supervises subordinates very closely (Q31)
My manager expects subordinates to follow his/her instructions precisely (Q33)
My manager believes that if he/she does not lay out goals and guidelines, subordinates will be passive and get nothing accomplished (Q41)
My manager expects subordinates to carry out his/her instructions immediately (Q49)
My manager is unwilling to spend time trying to improve poor performers (Q51)
After introducing new subordinates, my manager lets them make friends on their own (Q55)
My manager ´motivates´ subordinates by letting them know what will happen to them if their work is unsatisfactory (Q64)
Archetype 3
My manager works hard to ease tensions whenever they arise in my work group (Q1)
My manager requires subordinates to submit detailed reports of their activities (Q2)
My manager tries to reduce resistance to his/her decisions by telling subordinates what they have to gain (Q3)
My manager spends a lot of time reviewing subordinates´ progress to determine whether adjustments are necessary (Q6)
My manager works to develop close personal relationships with subordinates (Q8)
When subordinates fail at a task, my manager calmly but firmly lets them know why (Q10)
My manager spends time looking for opportunities for subordinates´ professional development (Q12)
My manager keeps everyone involved and well-informed about organizational issues that may affect them (Q13)
My manager discourages arguments that might lead to conflict among subordinates (Q14)
My manager often gives orders in the form of a suggestion, but makes it clear what he/she wants (Q15)
My manager discusses controversial changes in company policy at length with subordinates (Q21)
My manager encourages subordinates to talk to him/her about personal problems (Q22)
My manager praises subordinates for adequate work (Q23)
My manager believes subordinates´ feelings are as important as the task at hand (Q24)
My manager makes a special effort to explain to subordinates the purpose of their work (Q26)
My manager helps subordinates think through the who, when, and how of completing tasks (Q28)
My manager supervises subordinates very closely (Q31)
My manager holds frequent meetings to share information and ideas with subordinates (Q32)
When subordinates disagree with him/her, my manager explains why he/she wants something done a certain way (Q34)
My manager puts a great deal of effort into developing subordinates (Q36)
My manager relies on what he/she learns through personal contact with subordinates to use each person’s talent most effectively (Q37)
My manager believes that if he/she does not lay out goals and guidelines, subordinates will be passive and get nothing accomplished (Q41)
My manager relies on his/her knowledge and competence to influence subordinates (Q42)
My manager questions subordinates to understand why their goals are important to them (Q43)
My manager devotes a great deal of time to subordinates´ job security and fringe benefits (Q50)
My manager takes time to explain the reasons for decisions in terms of the best interests of the organization and his/her subordinates (Q52)
My manager often demonstrates concern for subordinates (Q53)
When making decisions, my manager tries to get a great deal of input from subordinates (Q54)
My manager spends a significant amount of time helping subordinates to improve their performance (Q56)
My manager frequently monitors subordinates´ progress on their tasks (Q58)
My manager often rewards performance that is adequate (Q60)
My manager almost always tells subordinates when they have done good work (Q65)
My manager encourages subordinates to participate in most decision making (Q68)

Associations with archetype(s) are only provided for behaviors with scores above the 80th percentile.

The comparison between the three archetypes and the six leadership styles is warranted, because we want to be sure that the three archetypes found are not merely a way to classify the six leadership styles in higher order categories. After all, this would then imply that the archetypes are just again some leadership styles that do not capture leaders as a person or configuration of behaviors. To further investigate the differences between the six leadership styles and our three archetypes, we therefore assigned all individual leaders in the sample to one of the three archetypes based on the weights estimated in the previous section and as reported in Table 2 and Figure 2. Two different cut-off points were used. First, we assigned each leader to the archetype for which he or she received the largest weight – that is, the archetype he or she was associated with the most. Second, we classified each leader only if he or she was of a “pure” type, meaning that they received a weight of more than 0.5 for one of the archetypes. The second classification was more conservative and was used as a robustness test. Based on the first classification, we correlate the three archetypes with the six leadership styles as present in the underlying dataset, see Table 3 for the results.

**Table 3:** Correlations between three archetypes, six leadership styles, and controls.

	Type 2	Type 3	Type 1	Gender	Native	Age	Tenure	Prim. School	Sec. Educ.	Some Univ.	Univ. Grad.	Adv. Degree	Direc-tive	Affil.	Author	Coach	Pace
Type 2																	
Type 3	−0.41																
Type 1	−0.483	−0.601															
Gender	−0.052	0.082	−0.034														
Native	−0.003	0.037	−0.033	0.024													
Age	−0.006	−0.135	0.135	−0.082	0.007												
Tenure	−0.027	−0.012	0.036	−0.068	0.066	0.457											
Primary School	−0.003	0.025	−0.022	0.022	0.021	0.035	0.057										
Secondary School	−0.002	0.039	−0.035	0.013	0.036	0.077	0.113	−0.029									
Some University	0.008	0.001	−0.008	0.005	0.002	−0.001	−0.007	−0.001	−0.003								
University Graduate	−0.006	0.017	−0.011	−0.009	0.038	−0.052	0.032	−0.087	−0.285	−0.009							
Higher Degree	0.008	−0.044	0.035	−0.002	−0.062	0.002	−0.107	−0.085	−0.278	−0.009	−0.824						
Directive	0.501	0.516	−0.933	0.022	0.041	−0.114	−0.027	0.015	0.026	0.009	0.014	−0.032					
Affiliative	−0.813	0.702	0.039	0.103	0.026	−0.062	0.015	0.016	0.028	−0.004	0.027	−0.046	−0.068				
Authoritative	−0.846	0.644	0.124	0.063	−0.002	−0.023	0.02	0.016	0.015	−0.005	−0.01	−0.001	−0.165	0.753			
Coaching	−0.578	0.865	−0.323	0.084	0.024	−0.121	−0.021	0.023	0.027	0	0.022	−0.042	0.221	0.705	0.68		
Pacesetting	0.375	−0.888	0.524	−0.077	−0.035	0.142	0.012	−0.028	−0.054	0.001	−0.02	0.057	−0.407	−0.61	−0.573	−0.729	
Participative	−0.782	0.534	0.172	0.026	0.023	0.001	0.032	−0.009	−0.008	−0.006	−0.01	0.016	−0.197	0.683	0.695	0.55	−0.452

*N* = 154,285. *p* < 0.001 for all coefficients.

Table 3 confirms that the six leadership styles are clearly but not exclusively associated with one of the three archetypes. Moreover, Table 3 demonstrates that the styles and archetypes are far from perfectly correlated.

As a more stringent test to investigate if the three archetypes overlap with (a combination of) the leadership styles, we applied regression analyses to compare the three archetypes with the six leadership styles. Note that the regressions are not meant to arrive at any causal relationships, but merely as a means to investigate whether and how the leadership styles and the archetypes are associated. For each archetype, we first regressed the archetype on the main or strongest correlated leadership style(s) of that archetype (see also Figure 3). The estimation results are shown in Table 4.

**Table 4:** Regression analyses for the three archetypes as a function of the six (interacted) leadership behaviors.

	Dependent variable					
		Type 2		Type 3		Type 1	
		(1)	(2)	(3)	(4)	(5)	(6)
Person.nSex	−0.022^***^ (0.001)	0.002^***^ (0.0002)	−0.0002 (0.001)	−0.001^***^ (0.0001)	0.005^***^ (0.001)	−0.001^***^ (0.0002)
Native	−0.009^***^ (0.001)	−0.002^***^ (0.0002)	0.008^***^ (0.001)	−0.001^***^ (0.0002)	−0.009^***^ (0.001)	0.003^***^ (0.0003)
Age	0.001^***^ (0.0001)	0.00001 (0.00001)	−0.001^***^ (0.00003)	0.00000 (0.00001)	0.001^***^ (0.0001)	−0.00002 (0.00002)
Tenure	−0.001^***^ (0.00005)	−0.00001 (0.00001)	0.0004^***^ (0.00003)	−0.00000 (0.00001)	0.0001^**^ (0.0001)	0.00001 (0.00001)
Factor(ceducationid) 2	0.007^*^ (0.004)	−0.001^*^ (0.001)	−0.003 (0.002)	−0.001 (0.001)	0.012^***^ (0.004)	0.002 (0.001)
Factor(ceducationid) 3	0.065^*^ (0.038)	−0.012^*^ (0.007)	0.021 (0.023)	−0.005 (0.006)	−0.139^***^ (0.043)	0.018 (0.011)
Factor(ceducationid) 4	0.013^***^ (0.004)	−0.003^***^ (0.001)	−0.013^***^ (0.002)	0.0001 (0.001)	0.020^***^ (0.004)	0.003^***^ (0.001)
Factor(ceducationid) 5	0.018^***^ (0.004)	−0.002^***^ (0.001)	−0.012^***^ (0.002)	0.002^**^ (0.001)	0.020^***^ (0.004)	0.0004 (0.001)
Directive	0.130^***^ (0.001)	1.578^***^ (0.125)		−1.716^***^ (0.104)		0.143 (0.204)
Affiliative		1.271^***^ (0.153)	0.046^***^ (0.001)	−2.237^***^ (0.127)		0.972^***^ (0.249)
Authoritative		0.888^***^ (0.144)	0.005^***^ (0.001)	−3.340^***^ (0.120)		2.458^***^ (0.235)
Coaching		1.526^***^ (0.175)	0.183^***^ (0.0005)	0.082 (0.145)		−1.614^***^ (0.286)
Pacesetting		1.191^***^ (0.111)		−1.542^***^ (0.092)	0.175^***^ (0.001)	0.354^**^ (0.180)
Participative		0.330^**^ (0.159)	0.002^***^ (0.001)	−2.324^***^ (0.132)		1.996^***^ (0.259)
Directive: Affiliative		−0.517^***^ (0.039)		0.604^***^ (0.032)		−0.089 (0.064)
Directive: Authoritative		−0.405^***^ (0.037)		0.895^***^ (0.031)		−0.492^***^ (0.061)
Affiliative: Authoritative		−0.375^***^ (0.036)		0.851^***^ (0.030)		−0.478^***^ (0.058)
Directive: Coaching		−0.575^***^ (0.043)		0.076^**^ (0.036)		0.500^***^ (0.070)
Affiliative: Coaching		−0.396^***^ (0.043)		0.402^***^ (0.035)		−0.006 (0.069)
Authoritative: Coaching		−0.323^***^ (0.042)		0.570^***^ (0.035)		−0.247^***^ (0.068)
Directive: Pacesetting		−0.449^***^ (0.028)		0.485^***^ (0.024)		−0.037 (0.046)
Affiliative: Pacesetting		−0.412^***^ (0.035)		0.598^***^ (0.029)		−0.187^***^ (0.058)
Authoritative: Pacesetting		−0.362^***^ (0.033)		0.879^***^ (0.028)		−0.518^***^ (0.054)
Coaching: Pacesetting		−0.499^***^ (0.042)		0.111^***^ (0.035)		0.389^***^ (0.069)
Directive: Participative		−0.366^***^ (0.041)		0.621^***^ (0.034)		−0.256^***^ (0.068)
Affiliative: Participative		−0.276^***^ (0.041)		0.608^***^ (0.034)		−0.334^***^ (0.067)
Authoritative: Participative		−0.184^***^ (0.040)		0.914^***^ (0.033)		−0.732^***^ (0.064)
Coaching: Participative		−0.126^***^ (0.048)		0.264^***^ (0.040)		−0.136^*^ (0.079)
Pacesetting: Participative		−0.272^***^ (0.034)		0.638^***^ (0.029)		−0.367^***^ (0.056)
Directive: Affiliative: Authoritative		0.135^***^ (0.009)		−0.215^***^ (0.008)		0.081^***^ (0.015)
Directive: Affiliative: Coaching		0.148^***^ (0.011)		−0.115^***^ (0.009)		−0.033^*^ (0.017)
Directive: Authoritative: Coaching		0.125^***^ (0.011)		−0.158^***^ (0.009)		0.033^*^ (0.017)
Affiliative: Authoritative: Coaching		0.091^***^ (0.009)		−0.184^***^ (0.007)		0.093^***^ (0.014)
Directive: Affiliative: Pacesetting		0.141^***^ (0.009)		−0.170^***^ (0.008)		0.030^*^ (0.015)
Directive: Authoritative: Pacesetting		0.121^***^ (0.009)		−0.243^***^ (0.007)		0.122^***^ (0.015)
Affiliative: Authoritative: Pacesetting		0.113^***^ (0.008)		−0.229^***^ (0.007)		0.116^***^ (0.014)
Directive: Coaching: Pacesetting		0.172^***^ (0.011)		−0.063^***^ (0.009)		−0.109^***^ (0.017)
Affiliative: Coaching: Pacesetting		0.131^***^ (0.011)		−0.143^***^ (0.009)		0.012 (0.017)
Authoritative: Coaching: Pacesetting		0.123^***^ (0.010)		−0.181^***^ (0.009)		0.058^***^ (0.017)
Directive: Affiliative: Participative		0.136^***^ (0.011)		−0.151^***^ (0.009)		0.015 (0.018)
Directive: Authoritative: Participative		0.107^***^ (0.010)		−0.227^***^ (0.009)		0.121^***^ (0.017)
Affiliative: Authoritative: Participative		0.082^***^ (0.009)		−0.205^***^ (0.007)		0.124^***^ (0.014)
Directive: Coaching: Participative		0.105^***^ (0.012)		−0.077^***^ (0.010)		−0.028 (0.020)
Affiliative: Coaching: Participative		0.050^***^ (0.011)		−0.118^***^ (0.009)		0.068^***^ (0.017)
Authoritative: Coaching: Participative		0.034^***^ (0.011)		−0.172^***^ (0.009)		0.137^***^ (0.017)
Directive: Pacesetting: Participative		0.127^***^ (0.009)		−0.177^***^ (0.008)		0.050^***^ (0.015)
Affiliative: Pacesetting: Participative		0.103^***^ (0.010)		−0.170^***^ (0.008)		0.068^***^ (0.015)
Authoritative: Pacesetting: Participative		0.091^***^ (0.009)		−0.249^***^ (0.008)		0.158^***^ (0.015)
Coaching: Pacesetting: Participative		0.097^***^ (0.012)		−0.115^***^ (0.010)		0.017 (0.019)
Directive: Affiliative: Authoritative: Coaching		−0.034^***^ (0.002)		0.048^***^ (0.002)		−0.014^***^ (0.004)
Directive: Affiliative: Authoritative: Pacesetting		−0.035^***^ (0.002)		0.060^***^ (0.002)		−0.026^***^ (0.004)
Directive: Affiliative: Coaching: Pacesetting		−0.045^***^ (0.003)		0.044^***^ (0.002)		0.001 (0.005)
Directive: Authoritative: Coaching: Pacesetting		−0.040^***^ (0.003)		0.053^***^ (0.002)		−0.012^***^ (0.004)
Affiliative: Authoritative: Coaching: Pacesetting		−0.034^***^ (0.002)		0.057^***^ (0.002)		−0.024^***^ (0.004)
Directive: Affiliative: Authoritative: Participative		−0.035^***^ (0.002)		0.049^***^ (0.002)		−0.014^***^ (0.004)
Directive: Affiliative: Coaching: Participative		−0.031^***^ (0.003)		0.031^***^ (0.002)		−0.001 (0.004)
Directive: Authoritative: Coaching: Participative		−0.025^***^ (0.003)		0.044^***^ (0.002)		−0.019^***^ (0.004)
Affiliative: Authoritative: Coaching: Participative		−0.013^***^ (0.002)		0.046^***^ (0.002)		−0.033^***^ (0.003)
Directive: Affiliative: Pacesetting: Participative		−0.039^***^ (0.003)		0.045^***^ (0.002)		−0.005 (0.004)
Directive: Authoritative: Pacesetting: Participative		−0.035^***^ (0.003)		0.064^***^ (0.002)		−0.029^***^ (0.004)
Affiliative: Authoritative: Pacesetting: Participative		−0.029^***^ (0.002)		0.057^***^ (0.002)		−0.029^***^ (0.003)
Directive: Coaching: Pacesetting: Participative		−0.042^***^ (0.003)		0.035^***^ (0.003)		0.007 (0.005)
Affiliative: Coaching: Pacesetting: Participative		−0.029^***^ (0.003)		0.043^***^ (0.002)		−0.014^***^ (0.004)
Authoritative: Coaching: Pacesetting: Participative		−0.028^***^ (0.003)		0.056^***^ (0.002)		−0.028^***^ (0.004)
Directive: Affiliative: Authoritative: Coaching: Pacesetting		0.011^***^ (0.001)		−0.016^***^ (0.0005)		0.005^***^ (0.001)
Directive: Affiliative: Authoritative: Coaching: Participative		0.007^***^ (0.001)		−0.011^***^ (0.0005)		0.004^***^ (0.001)
Directive: Affiliative: Authoritative: Pacesetting: Participative		0.010^***^ (0.001)		−0.014^***^ (0.0005)		0.005^***^ (0.001)
Directive: Affiliative: Coaching: Pacesetting: Participative		0.012^***^ (0.001)		−0.012^***^ (0.001)		0.001 (0.001)
Directive: Authoritative: Coaching: Pacesetting: Participative		0.011^***^ (0.001)		−0.015^***^ (0.001)		0.004^***^ (0.001)
Affiliative: Authoritative: Coaching: Pacesetting: Participative		0.008^***^ (0.001)		−0.015^***^ (0.0004)		0.007^***^ (0.001)
Directive: Affiliative: Authoritative: Coaching: Pacesetting: Participative		−0.003^***^ (0.0001)		0.004^***^ (0.0001)		−0.001^***^ (0.0002)
Constant	−0.235^***^ (0.005)	−2.231^***^ (0.504)	−0.529^***^ (0.003)	5.635^***^ (0.419)	−0.231^***^ (0.005)	−2.414^***^ (0.822)

Observations	154,285	154,285	154,285	154,285	154,285	154,285
Adjusted *R*^2^	0.259	0.975	0.767	0.985	0.279	0.948
F Statistic	6,005.615^***^ (df = 9; 154,275)	83,143.110^***^ (df = 71; 154,213)	42,219.360^***^ (df = 12; 154,272)	145,070.100^***^ (df = 71; 154,213)	6,645.062^***^ (df = 9; 154,275)	39,487.490^***^ (df = 71; 154,213)

^*^p < 0.1; ^**^p < 0.05; ^***^p < 0.01.

For Archetype 2, we explore the separate effect of the style with the strongest correlation in column 1, in this case directive leadership. For Archetype 3 we do the same in column 3 but now with authoritative, affiliative, coaching and participative leadership, and for Archetype 1 we explore the effect of pacesetting leadership in column 5. Secondly and crucially, we perform a full regression analysis for each of the archetypes (in columns 2, 4 and 6 for Archetypes 2, 3 and 1 respectively), where not only all leadership styles are included, but also all possible interaction effects between the six leadership styles. To do so, we include up to a maximum of a six-way interaction between the leadership styles.

The main take away from columns 1, 3, and 5 is that the leadership styles are all significantly associated with their respective archetype, that is to say the archetype that has the strongest correlation. But crucially, although these styles explain some of the variation in the archetypes, as can be derived from the R2, we can also conclude that the variance explained is far from perfect. The R2 value ranges from.26 for Archetype 2, to.27 for the Archetype 1, and.77 for Archetype 3.

In the consecutive columns 2, 4, and 6 of Table 4, each time we add not only the most relevant leadership style(s) as predictors, but also all possible interaction variables between the leadership styles, up to a maximum of a six-way interaction. With such an extensive set of interaction effects it becomes hard to interpret the sign of the coefficients (Jaccard and Turrisi, 2003), but interpretation is *not* the aim of these estimations. In line with the idea that each leader can be described as a configuration of leadership behaviors, the results of these regression models for the three archetypes show that almost all possible interactions are significant predictors of the archetypes.

Finally, we also checked whether the three archetypes follow from our dataset when we control for country culture. To do so, we re-did the archetypal analysis for each of the 11 country culture clusters as identified by Ronen and Shenkar (2013). With the possible exception of the Confucian cluster, all country culture clusters are indeed best characterized by the three archetypes that were found in the full sample (results not shown here because of brevity, but are available upon request).

### 4.2. One possible interpretation of the three archetypes

The results presented above allow us to give meaning to the content of these three archetypes. Archetype 1 contains five items and scores mainly high on the questions related to pacesetting leadership and on one item of participative leadership. Archetype 2 scores on items mainly related to directive leadership, but also to two items of pacesetting leadership. Finally, archetype 3 scores high and exclusively on affiliative, authoritative and coaching leadership. Moreover, it also scores high on items belonging to directive and to participative leadership. Clearly, these three archetypes are a configuration of leadership behaviors that are associated with various leadership styles.

The follow-up question is therefore, whether these kinds of archetypes can be given a meaningful interpretation. Following Fischer and Sitkin (2022), we build on the configurational tradition in adjacent fields. Examples from these fields indicate that, in order to arrive at meaningful configurations, it is important to search for relevant conceptual dimensions. For instance, Mintzberg (1980) five organizational structures were based on two key dimensions, namely coordinating mechanisms and design parameters (Fischer and Sitkin, 2022). Likewise, Cardinal et al. (2010) also used two dimensions to arrive at four organizational control types. To be clear, the aim of our paper is to investigate whether we can arrive at meaningful configurations of leadership behaviors. By selecting two constituent dimensions that allow us to determine a meaningful content for each of the three archetypes, we want to show that it is possible to do so, thereby using relevant insights form adjacent fields.

If we look at the content of the items that are linked to the three archetypes, in our view these three archetypes differ primarily along two dimensions: (a) the amount of time that managers spend with their employees, and (b) how managers communicate with subordinates (see below). Managers that are characterized as Archetype 1 do neither transmit nor receive information, nor do they intervene unless the need to do so arises. The main dimension here is the amount of time spent by managers with their subordinates, which is for this archetype rather minimal. The way of communication between managers and subordinates is less clear. Archetype 2 leaders primarily transmit information to subordinates by clearly telling them what is expected, preferably through rigid instructions, and by supervising them closely. Archetype 3 leaders on the one hand transmit information to subordinates through instructions, monitoring, and day-to-day management, which is represented by the overlap of questions 2, 31, and 41 with Archetype 2. But on the other hand, they also receive information, as opposed to Archetype 2 leaders, by spending time listening to subordinates’ comments, suggestions and disagreements, and trying to respond to these. Based on the above categorization of the three archetypes, one could label managers of Archetypes 1, 2, and 3 as “minimal,” “one-way,” and “two-way” leaders, respectively.

The classification of archetypes *via* the two dimensions as suggested above can be underpinned by research on communication styles and time allocation by managers. The two dimensions are at home in research in various sub-fields, notably communication studies, psychology, management as well as economics. De Vries et al. (2009, 2010) show for instance how various communication styles matter. The latter study shows specifically for leaders how their communication styles matter for leader effectiveness in terms of subordinates’ work engagement or job satisfaction.

When it comes to the time allocation of managers, and while using insights from social psychology, Penfield (1974) already wrote a seminal study on the time allocation of managers and a classification of what it is that managers actually do. The time allocation combined with the actual content of managerial activities is also the subject of recent research by Bandiera et al. (2020), who studied the behavior of 1,114 CEOs in six countries using CEO diary data. This study concludes that CEOs can indeed be split along the two dimensions communication style and content.

This notion about the relevance of (measuring) actual managerial communication and time allocation goes back to the seminal research by Mintzberg (1973) and it can be used to argue that the two dimensions alluded to above, communication style as well as time spent with subordinates, make sense (see for instance Luthans et al., 1985). What we take away from this literature is that the two dimensions identified can not only be used to demarcate but could also be used as conceptual building blocks for the three archetypes found.

## 5. Conclusion and discussion

Answering calls from leadership scholars to critically assess the use of leadership styles (Yukl, 1999; Dansereau et al., 2013; Van Knippenberg and Sitkin, 2013; Antonakis and House, 2014; Banks et al., 2016; Fischer and Sitkin, 2022), the aim of our paper is to investigate whether one can arrive, through archetypal analysis, at meaningful configurations of leadership behaviors. By employing the technique of archetypal analysis, we find ourselves in the company of other management scholars (e.g., Venaik and Midgley, 2015) who have already shown how archetypal analysis can be used in related fields of management. Our paper is, however, the first to apply archetypal analysis to leadership research, and the first to establish configurations of leadership for a large dataset (Fischer and Sitkin, 2022).

The results of our archetypal analysis among more than 150,000 leaders show two main findings. First, building on a set of 46 behavioral items measuring six leadership styles in total, the data show clear evidence for the existence of three archetypes of leadership behavior. Although the archetypes found do, to some extent, resemble one or more of the six leadership styles, they do not coincide with them, which shows that a configurational approach to leadership via archetypal analysis leads to a different classification of leaders than the standard variable oriented approach of leadership styles.

Second, the three resulting archetypes can be classified along two dimensions in our view, namely (a) the amount of time managers spend on interacting with their subordinates and (b) the communication style of the managers. Based on these two dimensions, we distinguish three types of leaders as “minimal,” “one-way” and “two-way” leaders. Managers that are characterized as “minimal” do neither transmit nor receive information, nor do they intervene unless the need to do so arises. The “one-way” leader primarily transmits information to subordinates by clearly telling them what is expected from subordinates, preferably through rigid instructions, and by supervising them closely. The “two-way” leader transmits information to subordinates through instructions, monitoring, and day-to-day management, and also receives information by spending time listening to subordinates’ comments, suggestions and disagreements, and trying to respond to these.

The first contribution of our paper is that it offers support for a configurational or person-oriented approach to leadership *via* the application of archetypal analysis. In doing so, we take as our starting point that leaders are not *a priori* reduced to showing only a limited number of leadership styles. Our results confirm that archetypal analysis indeed is a promising tool to arrive at meaningful configurations of leadership behaviors. More generally, our paper is an example of how a configurational approach can “overcome the current impasse in leadership research” (Fischer and Sitkin, 2022, p. 65), because it offers an alternative method to group together individual leadership behaviors, thereby circumventing the sketched problems with existing leadership styles that we described in the introduction.

As a follow-up, our second contribution is that we show that the archetypes found really differentiate from the well-established leadership styles in our dataset. To address this issue, we do not only confront the three archetypes with the underlying leadership styles but also with all possible interactions between these styles. These results provide further support for the person-oriented approach as a promising alternative to the variable-centered approach, which is still more common in leadership research. In addition, such a person-oriented and hence more holistic approach seems more fitting in a context where change and complexity are pressing and prominent.

The final contribution concerns the meaningfulness of our archetypes. We show that archetypal analysis, at least for our data set, results in three conceptually meaningful archetypes of leaders, when we classify them based on two conceptual dimensions (Fischer and Sitkin, 2022). These two dimensions, namely time-spending and communication style, have support in management research more broadly, going back to scholars like Mintzberg (1973) and Penfield (1974), but also following more recent work by De Vries et al. (2009, 2010) and Bandiera et al. (2020). Crucially, these two characteristics have previously not been identified as distinctive meta-features in relation to leadership behaviors. When it comes to future research, a key question would be whether these two features are also to be found in other data sets where different leadership behaviors are measured.

Our study has two main limitations. An important limitation is that we could not confront our archetypes with objective outcome measures. This is due to data limitations, but certainly a next (and necessary) step to further analyze the relevance of archetypes for leadership research. In our dataset, we do have access to subjective team outcome variables, such as team cohesiveness (Dion, 2000). Although these variables, being single source data, have various concerns in terms of endogeneity (Antonakis, 2017), we explored the relationship between our three archetypes and this outcome variable (results not shown here, but available upon request). We find that the three archetypes significantly explain variation in this outcome variable over and above the individual leadership styles. In particular, compared to the other two archetypes, the “two-way” archetype is significantly and positively related to team cohesiveness. This result suggests that especially leaders who spend relatively more time to their employees, and communicate relatively more in an interactive manner, are associated with teams that are more cohesive and thereby more effective.

A second limitation is the possible meaning of the three found archetypes beyond our data set and analysis. We would like to stress that the archetype classifications as well as the two suggested dimensions as described here, are unique to this particular dataset and behavioral configuration, and thus should not be interpreted as a set of classifications that is definitive across all contexts. Similarly, but beyond the scope of the present paper, one could ask whether other clustering techniques like latent profile analysis or mixture models would yield similar classifications of leaders.

The generalizability of configurational findings like the ones shown in the present paper is a crucial issue for future research (Woo et al., 2018; Fischer and Sitkin, 2022). Therefore, we hope that our study on archetypes will serve as a motivation for other scholars to investigate other datasets, possibly in different contexts. The purpose is not only to test whether they would find comparable archetypes, but more importantly to further improve the validity of a configurational approach to leadership, and in doing so, also to compare those findings to other person or pattern oriented clustering techniques (e.g., Foti et al., 2012).

## Data availability statement

The data analyzed in this study is subject to the following licenses/restrictions: The authors do not have permission to share data. Requests with respect to the use of the dataset can be made to the corresponding author, who will inform the owner of the dataset, Korn Ferry, about this request.

## Ethics statement

Ethical review and approval was not required for the study on human participants in accordance with the local legislation and institutional requirements. Written informed consent for participation was not required for this study in accordance with the national legislation and the institutional requirements.

## Author contributions

JS, HG, DS, and TV contributed to conception and design of the study and wrote sections of the manuscript. DS organized the database. DS and TV performed the statistical analysis. JS and HG wrote the first draft of the manuscript. All authors contributed to manuscript revision, read, and approved the submitted version.

## Conflict of interest

The authors declare that the research was conducted in the absence of any commercial or financial relationships that could be construed as a potential conflict of interest.

## Publisher’s note

All claims expressed in this article are solely those of the authors and do not necessarily represent those of their affiliated organizations, or those of the publisher, the editors and the reviewers. Any product that may be evaluated in this article, or claim that may be made by its manufacturer, is not guaranteed or endorsed by the publisher.
